# Inverse association of fat mass, but not lean mass, with glycated albumin in hemodialysis patients with or without diabetes mellitus

**DOI:** 10.1080/0886022X.2019.1659819

**Published:** 2019-09-09

**Authors:** Jiro Miyawaki, Senji Okuno, Katsuhito Mori, Eriko Nishio, Kyoko Norimine, Masafumi Kurajoh, Tomoyuki Yamakawa, Shigeichi Shoji, Masaaki Inaba

**Affiliations:** a Kidney Center, Shirasagi Hospital, Osaka, Japan;; b Department of Nephrology, Osaka City University Graduate School of Medicine, Osaka, Japan;; c Department of Metabolism, Endocrinology and Molecular Medicine, Osaka City University Graduate School of Medicine, Osaka, Japan

**Keywords:** Glycated albumin, fat mass, hemodialysis, diabetes mellitus

## Abstract

**Background:** Glycated albumin (GA), which is independent of anemia and/or use of erythropoiesis-stimulating agents, might provide a more precise measure than glycated hemoglobin (HbA1c) in hemodialysis patients. The present study examines whether body composition is associated with GA besides glycemic control in hemodialysis patients.

**Methods:** This study included 90 hemodialysis patients with diabetes mellitus (DM) and 86 hemodialysis patients without DM. We examined blood parameters after an overnight fast and body fat and lean mass using dual X-ray absorptiometry 21–24 h after completing the dialysis session.

**Results:** The mean body mass index (BMI) was 22.0 kg/m^2^. BMI and truncal fat mass were significantly higher, and total fat mass tended to be higher in hemodialysis patients with DM than in those without DM. GA exhibited inverse correlations with BMI, total lean mass, total fat mass, and truncal fat mass in hemodialysis patients with and without DM; however, there was a lack of correlation with total lean mass in patients without DM. In multiple regression analysis including total fat mass and total lean mass simultaneously as independent variables, total fat mass (with DM: *β* = –0.322, *p* = .006) (without DM: *β* = –0.391, *p* < .001), but not total lean mass, in addition to log fasting plasma glucose, emerged as an independent factor associated with GA in hemodialysis patients with and without DM. When total fat mass was replaced with truncal fat mass (with DM: *β* = –0.311, *p* = .007) (without DM: *β* = –0.396, *p* < .001), the association remained significant and independent with GA in both patient groups.

**Conclusions:** Higher total fat mass, particularly truncal fat mass, might be associated with lower GA levels, beside glycemic control, in hemodialysis patients with or without DM.

## Introduction

Strict glycemic control prevents the development of diabetic complications in patients with diabetes mellitus (DM) [1–3], and measurement of glycated protein in the blood is considered essential to estimate integrated glycemic control in patients with DM. Previous reports, including our report [4], established that glycated albumin (GA) might allow more precise measurement than HbA1c in hemodialysis patients [5] as hemodialysis patients who are mostly treated with erythropoiesis-stimulating agents (ESAs) show falsely lower HbA1c levels mainly owing to an increased proportion of young erythroblasts in circulation [6]. Furthermore, GA might be superior to HbA1c as a glycemic marker in hemodialysis patients as GA might represent postprandial glucose excursion, which is a characteristic of hemodialysis patients with DM without postprandial urinary glucose excretion [7].

The clinical significance of GA as a glycemic marker is evident, particularly in hemodialysis patients with DM, not only from its precision of integrated glycemic control [4] and sensitive reflection of postprandial glucose excursion [8] but also from its precise prediction of mortality in hemodialysis patients with DM in our previous small-scale study [9], as well as in a previous large-scale epidemiological study [10].

However, it was recently reported that the GA level was significantly negatively associated with body mass index (BMI) in patients with DM [11]; although, most hemodialysis patients, either with or without DM, might not be obese enough to show a change in the GA level, as in patients with DM and without chronic kidney disease (CKD).

Based on this background, we examined (i) whether BMI is associated with the GA level independent of glycemic control in hemodialysis patients with or without DM, (ii) whether fat mass or lean mass is associated with the GA level, and (iii) which part of fat mass (total fat mass or truncal fat mass) might be associated with the GA level.

## Subjects and methods

### Subjects and study design

We conducted a cross-sectional observational study in 90 hemodialysis patients with DM and 86 hemodialysis patients without DM at Shirasagi Hospital, Osaka, Japan. The diagnosis of DM was based on a history of DM or the American Diabetes Association criteria [4]. We excluded patients with acute illness, infection, malignancy, thyroid dysfunction, and liver dysfunction. Each patient provided written informed consent before enrollment. This study was approved by the Ethics Review Committee of Shirasagi Hospital (approval no. H17-5) and was conducted in accordance with the principles of the Declaration of Helsinki.

### Laboratory measurements

Blood samples were obtained just before the start of the morning hemodialysis session after an overnight fast. Hemoglobin, fasting plasma glucose (FPG), and serum albumin and C-reactive protein (CRP) levels were measured using routine laboratory methods. The GA level was measured using an enzymatic method with the Lucica GA-L kit (Asahi Kasei Pharma Corporation, Tokyo, Japan), as previously reported [12]. Briefly, GA was hydrolyzed to amino acids by albumin-specific proteinase and then oxidized by ketoamine oxidase to produce hydrogen peroxide, which was quantitatively measured. The GA level was calculated as the percentage of GA relative to total albumin, which was measured using the new bromocresol purple method [13]. The GA assay is not influenced by the physiologic levels of ascorbic acid, bilirubin, and glucose up to 1000 mg/dL.

### Measurement of body composition using dual X-ray absorptiometry

Body weight in this study refers to ‘dry weight’. Body fat mass was measured using dual X-ray absorptiometry (DXA; QDR-4500; Hologic, Waltham, MA, USA), as previously described [14–16]. The reproducibility of fat mass measurements using DXA was less than 2%, as previously reported [15]. Lean mass was calculated as fat-free mass by subtracting fat mass from dry weight to avoid the possible influence of body fluid excess on lean mass measured using DXA. A previous study reported that fat mass assessed using DXA did not change following one session of hemodialysis, whereas DXA-measured lean mass, excluding bone, showed a significant decrease, as predicted by body weight reduction on water removal [17]. BMI was defined as the patient’s weight in kilograms divided by the square of the patient’s height in meters.

### Statistical analysis

Data are expressed as number, mean ± standard deviation, or median (interquartile range). Simple regression analysis was performed to examine the relationships among BMI, total fat mass, total lean mass, truncal fat mass, and GA. We used Spearman’s coefficient to assess the correlation between FPG and GA. As the distributions of CRP and FPG were skewed, they were log-transformed for multiple regression analysis to obtain a normal distribution. Multiple regression analysis was performed to examine the influence of age, sex, hemodialysis duration, serum albumin, log CRP, log FPG, total fat mass, total lean mass, and truncal fat mass. All statistical analyses were performed using Statview version 5.0 software (SAS Institute Inc., Cary, NC, USA). A *p* value of <.05 was considered statistically significant.

## Results

### Clinical characteristics of hemodialysis patients with and without DM

Table 1 shows the clinical characteristics of the 90 hemodialysis patients with DM and 86 hemodialysis patients without DM included in this study. The FPG and GA levels in hemodialysis patients with DM were 126 (range, 99–161) mg/dL and 23.6%±7.4%, respectively, while the respective values in hemodialysis patients without DM were 83 (77–89) mg/dL and 15.2%±2.0%. The levels were higher in hemodialysis patients with DM than in those without DM. In addition, BMI and truncal fat mass were significantly higher in hemodialysis patients with DM than in those without DM. The total fat mass tended to be higher in hemodialysis patients with DM than in those without DM, without statistical significance.

**Table 1.** Clinical characteristics of hemodialysis patients with and without DM.

	Total (*n* = 176)	With DM (*n* = 90)	Without DM (*n* = 86)	*p* Value
Age (years)	68.2 ± 11.1	67.2 ± 10.1	69.2 ± 12.1	.248
Sex, male/female (*n*)	100/76	51/39	49/37	.999
HD duration (years)	7.0 ± 6.0	5.2 ± 4.4	8.9 ± 6.9	<.001
Height (cm)	157 ± 9	158 ± 10	157 ± 9	.522
Weight (kg)	55.5 ± 12.5	57.4 ± 12.2	53.4 ± 12.5	.033
BMI (kg/m^2^)	22.3 ± 4.1	22.9 ± 4.1	21.5 ± 4.0	.021
Albumin (g/dL)	3.6 ± 0.3	3.6 ± 0.3	3.7 ± 0.3	.323
Hemoglobin (g/dL)	10.7 ± 1.0	10.8 ± 1.0	10.6 ± 1.0	.248
CRP (mg/L)	1.6 (0.7–4.2)	1.5 (0.5–3.6)	1.7 (0.9–5.3)	.512
FPG (mg/dL)	93 (82–127)	126 (99–161)	83 (77–89)	<.001
GA (%)	19.5 ± 6.8	23.6 ± 7.4	15.2 ± 2.0	<.001
Total fat mass (kg)	15.7 ± 7.2	16.7 ± 7.1	14.6 ± 7.1	.053
Total lean mass (kg)	36.8 ± 7.5	37.4 ± 7.6	36.1 ± 7.5	.237
Truncal fat mass (kg)	8.1 ± 4.2	8.9 ± 4.3	7.2 ± 3.9	.008

DM: diabetes mellitus; HD: hemodialysis; BMI: body mass index; CRP: C-reactive protein; FPG: fasting plasma glucose; GA: glycated albumin.

Data are presented as mean ± standard deviation, number, or median (interquartile range).

### Correlations of GA with BMI, total lean mass, total fat mass, and truncal fat mass in hemodialysis patients with or without DM

**Figure 1.**
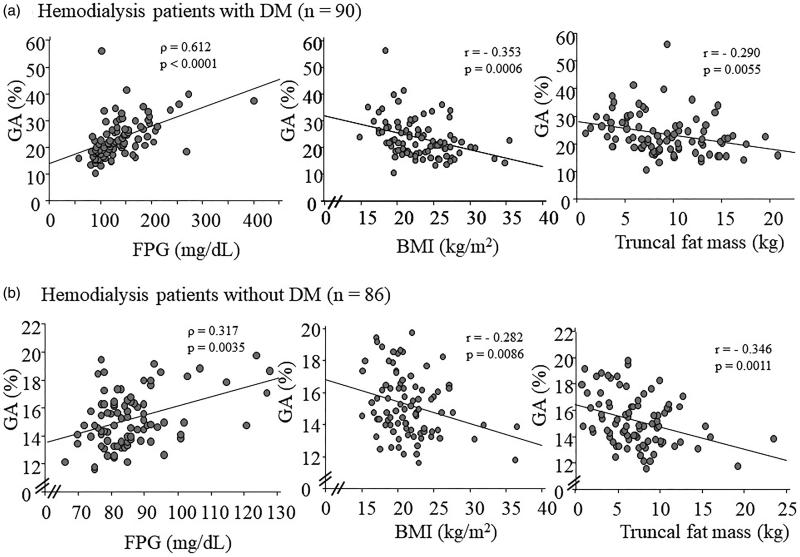
Correlations of GA with FPG, BMI, and truncal fat mass in hemodialysis patients with and without DM. GA exhibited significant and inverse correlations with FPG (left panel), BMI (middle panel), and truncal fat mass (right panel) in hemodialysis patients with DM (a) and without DM (b). GA: glycated albumin; FPG: fasting plasma glucose; BMI: body mass index; DM: diabetes mellitus.

GA exhibited significant and inverse correlations with the parameters of body composition, BMI, total lean mass, total fat mass, and truncal fat mass in hemodialysis patients with or without DM; however, there was no significant correlation of GA with total lean mass in hemodialysis patients without DM. Figure 1 shows significant correlations of GA with FPG (left panel), BMI (middle panel), and truncal fat mass (right panel) in hemodialysis patients with and without DM.

### Multiple regression analysis of factors for GA in hemodialysis patients with and without DM

Table 2 presents the results of multiple regression analysis of various clinical variables to evaluate their independent association with GA in hemodialysis patients with and without DM. In hemodialysis patients with DM, model 1, which included total fat mass and total lean mass, in addition to age, sex, hemodialysis duration, serum albumin, log CRP, and log FPG as independent variables, total fat mass and log FPG were significant and independent factors associated with GA. In model 2, which included truncal fat mass in the place of total fat mass, truncal fat mass was a significant and independent factor associated with GA. Total lean mass was not associated with GA in either model 1 or model 2. In hemodialysis patients without DM, total fat mass and truncal fat mass, in addition to log FPG, were significantly associated with GA. Moreover, age was significantly positively associated with GA in hemodialysis patients without DM.

**Table 2.** Multiple regression analysis to elucidate the total and truncal fat mass independently associated with GA in hemodialysis patients with and without DM.

	Total (*n* = 176)				With DM (*n* = 90)				Without DM (*n* = 86)			
	Model 1		Model 2		Model 1		Model 2		Model 1		Model 2	
	*β*	*p* Value	*β*	*p* Value	*β*	*p* Value	*β*	*p* Value	*β*	*p* Value	*β*	*p* Value
Age (years)	–0.053	.396	–0.053	.410	–0.112	.264	–0.107	.288	0.322	.005	0.315	.006
Sex (male 1, female 0)	–0.003	.966	0.011	.892	–0.003	.985	–0.012	.930	–0.222	.105	–0.191	.149
HD duration (years)	–0.090	.104	–0.088	.114	–0.069	.443	–0.063	.479	–0.193	.034	–0.183	.042
Albumin (g/dL)	–0.018	.766	–0.017	.783	0.022	.820	0.032	.745	0.202	.064	0.195	.073
Log (CRP (mg/L))	0.126	.027	0.123	.032	0.233	.013	0.229	.014	0.235	.017	0.231	.018
Log (FPG (mg/dL))	0.687	<.001	0.694	<.001	0.451	<.001	0.447	<.001	0.343	<.001	0.358	<.001
Total lean mass (kg)	–0.089	.290	–0.099	.234	–0.209	.109	–0.225	.080	0.198	.177	0.193	.183
Total fat mass (kg)	–0.185	.004	–	–	–0.322	.006	–	–	–0.391	<.001	–	–
Truncal fat mass (kg)	–	–	–0.172	.006	–	–	–0.311	.007	–	–	–0.396	<.001
*R*^2^	0.523		0.521		0.368		0.366		0.380		0.386	

DM: diabetes mellitus; GA: glycated albumin; HD: hemodialysis; CRP: C-reactive protein; FPG: fasting plasma glucose.

## Discussion

The present cross-sectional observational study demonstrated that the GA level was significantly and inversely associated with BMI, total fat mass, and truncal fat mass, but not with total lean mass, in hemodialysis patients with and without DM (Table 2). These findings suggested that higher fat mass, particularly in the truncal region, might be associated with lower GA levels, which are independent of glycemic control, in hemodialysis patients with or without DM. Although it was previously reported that higher BMI might be associated with lower GA levels in obese patients with DM and without CKD [11], the present study demonstrated a significant and inverse association of total and truncal fat mass with GA in patients with and without DM on maintenance hemodialysis. Although hemodialysis patients do not often exhibit visceral obesity, as reflected by a BMI of approximately 22 kg/m^2^ among the enrolled hemodialysis patients (Table 1), the present study findings raise the concern that hemodialysis patients with DM, including those without visceral obesity, might exhibit lower GA levels for their glycemic control.

Our previous study [4] and studies by other researchers [5,18] have demonstrated the clinical utility of GA over HbA1c in hemodialysis patients, as renal anemia and use of ESAs, which are required by more than 90% of chronic hemodialysis patients to maintain their hemoglobin levels within the target range, suppress the HbA1c value independent of glycemic control, leading to the underestimation of glycemic control in such patients. GA is superior to HbA1c as a marker for glycemic control because it is not affected by serum albumin, anemia, or ESA usage [19]. Thus, GA is a popular glycemic marker in Japanese hemodialysis patients [20]. Furthermore, we previously reported that GA, rather than HbA1c, might provide better assessment of the progression of atherosclerosis as assessed by pulse wave velocity [21] and medial arterial calcification [22] in hemodialysis patients. This is consistent with our recent report showing that GA might more precisely reflect postprandial glucose excursion, which is a definite risk factor for atherosclerosis [7].

Till date, it has been established that serum GA levels are influenced by several factors associated with the altered half-life of serum albumin independent of glycemic status [23]. For example, in pre-dialysis patients with DM and CKD, overt proteinuria decreases GA levels independent of glycemic control, probably owing to the shorter half-life of albumin in circulation [24]. Furthermore, it has been reported that increased thyroid function is significantly correlated with lower GA levels because of the promotion of albumin catabolism by the thyroid hormone [25]. In addition, it has been reported that serum GA is positively associated with age [24,26].

A previous study demonstrating that serum GA might be inversely correlated with BMI was performed in obese patients with DM and without CKD [11]. The mechanism was explained by hyperinsulinemia resulting from obesity that might increase the rate of albumin catabolism. This notion is consistent with the finding of the present study indicating that higher truncal fat mass, but not lean mass, was significantly associated with lower GA levels as hyperinsulinemia occurs as a result of visceral obesity. Furthermore, it was reported that increased visceral adiposity might induce inflammation, which might not only increase the rate of albumin catabolism but also reduce the rate of albumin synthesis [27,28]. This notion was supported by data showing a significant association of GA with high-sensitivity CRP in such patients. Another unconfirmed possibility may be the different turnover of GA compared to non-glycated one under some condition such as visceral adiposity.

GA is widely used as an indicator for glycemic control in hemodialysis patients in Japan [29]. GA might be a more reliable glycemic marker than HbA1c in most cases [29]. However, there are a few unexceptional cases who show unexpected low GA levels in daily clinical practice. One of confounding factors might be adiposity as shown in this study. Therefore, in patients showing a GA level significantly lower for their PG level, care should be taken when interpreting the GA data.

The present study has some limitations. All patients were Japanese and the sample size was relatively small. Therefore, the association of GA with BMI or truncal fat mass with GA should be confirmed in a large-scale study. Furthermore, the association between two parameters identified in Japanese patients might not be applicable to patients of other ethnicities. Finally, we identified lean mass by subtracting fat mass from dry weight. Previous report suggested that ultrafiltration could mainly remove extracellular fluid compared to intracellular one in hemodialysis patients [30]. If dry weight was accomplished, the identification of lean mass might be acceptable. However, it has been reported that 17.2% of hemodialysis patients had intradialytic hypotension [31]. Importantly, the presence of diabetes was significantly associated with this adverse event. In this study, some patients, especially diabetic patients, might not be able to reach the planned dry weight. In such cases, lean mass might possibly reflect considerable fluid retention, to various degrees.

In conclusion, it was demonstrated that higher BMI, particularly involving higher truncal fat mass, is associated with lower GA levels in hemodialysis patients with or without DM. This association may necessitate the consideration of truncal fat mass before interpretation of GA levels in hemodialysis patients with DM when the GA levels appear to be low for glycemic control.
